# Strengths-Based Approach for Mental Health Recovery

**Published:** 2013

**Authors:** Huiting Xie

**Affiliations:** 1 Senior Staff Nurse, Nursing Training, Institute of Mental Health, Buangkok View, Singapore.

**Keywords:** Community, Mental Health, Mental Health Recovery, Mental Illnesses, Strengths-Based Approach

## Abstract

Many health systems have traditionally adopted a view of mental disorders based on pathologies and the risk individuals have towards mental disorders. However, with this approach, mental disorders continue to cost billions a year for the healthcare system. This paper aimed to introduce and explore what the strengths-based approach is in the psychiatric arena. Strengths-based approach moves the focus away from deficits of people with mental illnesses (consumers) and focuses on the strengths and resources of the consumers. The paper also aligned the relevance of strength-based approach to mental health nursing and its contribution to mental health recovery.

**Declaration of interest:** None.

## Introduction

Mental health problems have become one of the major priorities for legislators and policy makers. Mental disorders cost the United States’ healthcare system billions of dollars per year. The cost of lost employment or decreased productivity and social welfare programs have been estimated at USD 273 billion a year of which about USD 70 billion is the estimated cost of untreated mental illnesses (1). According to a study by the World Health Organization (WHO) in 2001, mental illnesses rank first in terms of causing disability as compared to other diseases (2). Many health systems have traditionally adopted a medical view of mental disorders based on diagnostic categories such as ‘schizophrenia’, ‘bipolar disorder’ and ‘personality disorders’. The emphasis has been on looking for pathologies or symptoms in people with mental illnesses based on the diagnostic criteria for mental disorders.

In 1979, the term “salutogenesis” was coined by Aaron Antonovsky, a professor of medical sociology, to go beyond looking at disease to focus on factors that support human health and well-being (3). The strengths-based approach does so by focusing the attention on individuals’ attributes that promote health, instead of focusing on symptoms and pathologies that induce sickness.

This paper started with a review of the social and economic challenges in the mental health arena to introduce the context for strengths-based approach. This paper also drew some beginning parallels of the strength-based approach to mental health recovery and present supporting evidence from the literature. A process for practitioners to engage in strengths-based approach was also presented. People with mental illnesses were addressed as consumers in this paper.

**Why the strengths-based approach?**

Traditionally, the mental health arena is highly influenced by the medical model where severe mental illnesses, are considered chronic with irreversible neuropathological brain changes and information-processing deficits (4). Mental health recovery seems like an impossible dream.

As healthcare providers paint a gloomy picture of people with mental illnesses (consumers), consumers also view themselves in a negative light. They often realize that they are different from others. They may isolate themselves, which per se affect their self-esteem. Researchers found that 24% of the people with schizophrenia scored low on self-esteem on the Rosenberg self-esteem scale (RSES) (5). In consumers with low self-esteem, compromised quality of life (QoL) and poor psychosocial functioning are often observed (6, 7). Consumers with such negative self-appraisals perform badly in the community, and are more likely to relapse (8), thus impeding their recovery. 

Instead of employing the traditional medical model which emphasizes on pathology, focusing on problems and failures in people with mental illnesses; the strength-based approach allows practitioners to acknowledge that every individual has a unique set of strengths and abilities so that he/she can rely on to overcome problems (9). 

Helping consumers’ recovery has become the fundamental goal for mental health practitioners (10). Mental health recovery is a personal journey of gaining increasing meaningful life despite the presence of mental illnesses (11). To recover, consumers have to be confident that they have the ability to recover from mental health conditions. The strengths-based approach aligns itself with the notion of mental health recovery by focusing on person’s ability, helping them developing the confidence to embark on the journey of recovery and aiding them to progress towards mental health recovery. Attention is placed on people’s abilities rather than their shortcomings, symptoms or difficulties. Mental health issues are seen as a normal part of human life (12). Consumers’ positive attributes including assets, aspirations, hopes and interests are elicited and cultivated. According to Gable and Haidt (13), an understanding of strengths can help to prevent or lessen the damage of disease, stress and disorder. 

**Literature review on effectiveness of strengths-based approach**

It has been suggested that people have strengths within themselves that can contribute to recovery. Personal factors could aid the recovery process (14). A study on 55 consumers found that the presence of personality asset significantly predicted long term trend of improvement in disability over a follow-up period of 16 years (15). Additionally, in a large scale web-based retrospective study where 1008 participants considered themselves to have experienced serious psychological problems or emotional difficulties, the findings revealed that recovery from psychological disorders was associated with greater character strengths (16). 

Strengths have been linked to prediction of positive outcomes. In a study, providing the multi-disciplinary team with strength-based data resulted in better academic, social and overall outcomes for students with emotional and behavioral disorders as compared to traditional socio-emotive report that focused on the problems that students were facing (17). This suggests the possible usefulness of strength-based assessment. Indeed, in another study on children and adolescents living in residential homes, the level of strengths significantly predicted success in the reduction of risk behaviors (18). Even in the community, studies have shown the importance of focusing on strengths rather than deficits. Strengths assessments were associated with good behavioral functioning and greater competencies (19, 20).

Strengths-based approach also impacted life satisfaction. In a study by Rust et al. (21), 131 undergraduates were randomly assigned into the control group with no treatment or in one of the two treatment groups. One treatment group involved participants working on two strengths while participants in the other treatment group worked on a weakness and a strength point for a period of 12 weeks. The results showed no statistically significant differences in life satisfaction between the two treatment groups but the treatment groups had significantly higher life satisfaction scores than the control group. 

Findings from the literature have shown that individuals’ strengths are related to mental health improvement. These strengths can bring about positive outcomes in various aspects of life as satisfaction, functional status or health status, and have the potential to aid mental health recovery.

**Strengths-based approach in mental health practice**

Mental health care approaches in the community setting have moved in the direction towards encouraging people to cultivate their interests, identify and build their own strengths to pursue their goals (22). Policies, practice methods, and strategies have been created that identify and draw upon the strengths of individuals. Practitioners believe that the consumers have strengths that can be utilized for their recovery and work with consumers to facilitate the use of these strengths.

**Challenges**

It is often challenging for mental health practices to move from a pathology-based model to an individualized, strengths-based approach. Practitioners have been socialized to derive at a diagnosis by means of their education and training. The common perception is that an accurate diagnosis helps practitioners to institute the appropriate medical treatment to the consumers. Practitioners are often comfortable and confident in their role as expert. Strengths-based approach requires that practitioners acknowledge that they may not be all significant in the life of consumers. However, practitioners can use their knowledge to help consumers to utilize their strengths and integrate these into the recovery process.

In addition, many consumers may not seek services voluntarily and are often viewed as resistant or non-compliant. All these add up to interfere with the establishment of a process for the consumers and practitioners to identify and work on consumers’ strengths. In addition, consumers who are from the lower socio-economic group or who are experiencing stigma may not access mental health service at an optimal level. Furthermore, a lack of mental health resources coupled with large case-loads poses a major challenge to creating individualized strengths-based service plans. 

**Strategies**

The principles of strength-based approach were highlighted by Saleebey (23). Firstly, everyone possesses strengths that can be utilized to improve the quality of their life. Secondly, the consumer’s motivation to have better life stems from the focus on their strengths. And, finally, all environments contain resources that help consumers develop their strengths.

Consumers’ families and communities may be part of the resources consumers rely on to develop their strengths (23). Mental health practices may partner with local community organizations. These local organizations can help to identify and develop informal support system for the consumers or to provide facilities for the consumers to hold meetings or activities. Mental health practices can develop a formalized structure that requires participation from consumers as well as input from their families and communities. People with mental health conditions can use activities that they perceive as meaningful to aid their recovery (14). Meetings, educational sessions and social interventional gatherings between consumers, their family members and practitioners can be part of the formalized structure. Opportunities can be created for consumers to lead and share success stories with one another as well as practitioners and other partners involved. With a formalized structure, practitioners are better able to prioritize meetings between consumers, family and community among competing demands on their time. 

Therapeutic relationships may also be developed between the practitioners and consumers. The focus of therapeutic relationships does not lie in people’s diseases but rather in relationships that people have (24). The focus on strengths moves the practitioner away from the tendency to blame consumers, but towards discovering how people have strived despite adverse circumstance such as relapse of mental illnesses. The focus is upon the person’s strengths, desires, interests, aspirations, abilities, knowledge, and not on their deficits, weaknesses, problems or needs as seen by others. It does not disregard the real pain and struggles of people with mental illnesses but challenges the inadequacy of the sole focus of pathology (25). 

In such therapeutic relationships, practitioners and consumers are equal partners. The consumers’ autonomy is recognized. Consumers are the ones possessing the strengths and they will also be the ones using their strengths for their recovery. Consumers take the driver seat and their preferences are incorporated into the therapeutic relationships. The practitioner serves as a partner who has the professional and technical knowledge to facilitate consumers’ identification and utilization of their strengths to progress towards recovery. As in all partnerships, every party has a vested interest to help the other succeed. Consumers depend upon the practitioners for technical advice while the opinions of the consumers’ help practitioners understand them better. The recovery of the consumer re-affirms the efforts of the practitioners. The entire family and community may also be involved in the partnership. They could be potential resources to support the recovery of the consumers. 

Practitioners can begin the development of therapeutic relationships using the strengths-based approach through an assessment of consumer’s strengths. The strengths of each consumer are unique to the consumer. It can be almost anything dependent on circumstance; however, some capacities, resources, and assets commonly appear on a roster of strengths. These include: personal qualities, virtues and traits; what the person has learnt about themselves, others and the world; what people know about the world around them from education or life experience; the talents people have; cultural and personal stories, informal networks, institutions or professional entities (26, 27). Saleebey (23) suggested that strengths can be found by looking around for evidence of the person’s interests, talents and competencies and by listening to their stories. Epstein et al. (28) suggested chatting informally with consumers to help elicit their strengths. If the consumers have difficulties identifying their strengths, practitioners might chat with the consumers asking general questions about their hobbies or activities so that they enjoy doing, or how they have gotten through the acute phase of their illnesses in the past. The consumers may also be asked to think about their achievements at work, school or personal life. From the identified strengths, practitioners work with consumers to identify strengths that can be utilized to help them deal with their issues at the moment. After which a plan may be developed to utilize the strengths with the consumer setting the goals of the plan and deriving at the planned details. 

This may mean a significant shift for practitioner, focusing on issues that the consumers identify as important, rather than what the practitioners perceive to be important. The consumers are asked about the challenges they are facing and what issues need to be resolved first. The plan can then be implemented; for example, by asking the consumers to use one or more of their identified strength/s every day for one week. Consumers can follow-up with the practitioners a week later to talk about the approach and this can be an opportunity for the practitioners to evaluate consumers’ progress.

## Conclusion

The practice of mental health has been shaped largely by the medical model where the focus has been on solving problems and controlling the symptoms of mental illnesses. The strength based approach focuses on the positive aspect on consumers. Identification and utilization of the strengths consumers have could put them on the road to recovery and nursing with its emphasis of caring and individual centered approach are in the position to endorse strength-based approach. Creation of partnership among consumers, clinicians and other community agencies as well as implementing certain policies and practices may minimize or overcome potential challenges associated with strengths-based approach.
